# Predicting virus-host association by Kernelized logistic matrix factorization and similarity network fusion

**DOI:** 10.1186/s12859-019-3082-0

**Published:** 2019-12-02

**Authors:** Dan Liu, Yingjun Ma, Xingpeng Jiang, Tingting He

**Affiliations:** 1 School of Computer, Central China Normal University, Wuhan, Hubei China; 2 Hubei Provincial Key Laboratory of Artificial Intelligence and Smart Learning, Central China Normal University, Wuhan, Hubei China

**Keywords:** Virus-host association, Logistic matrix factorization, Similarity network fusion, Oligonucleotide frequency, Gaussian interaction profile

## Abstract

**Background:**

Viruses are closely related to bacteria and human diseases. It is of great significance to predict associations between viruses and hosts for understanding the dynamics and complex functional networks in microbial community. With the rapid development of the metagenomics sequencing, some methods based on sequence similarity and genomic homology have been used to predict associations between viruses and hosts. However, the known virus-host association network was ignored in these methods.

**Results:**

We proposed a kernelized logistic matrix factorization with integrating different information to predict potential virus-host associations on the heterogeneous network (ILMF-VH) which is constructed by connecting a virus network with a host network based on known virus-host associations. The virus network is constructed based on oligonucleotide frequency measurement, and the host network is constructed by integrating oligonucleotide frequency similarity and Gaussian interaction profile kernel similarity through similarity network fusion. The host prediction accuracy of our method is better than other methods. In addition, case studies show that the host of crAssphage predicted by ILMF-VH is consistent with presumed host in previous studies, and another potential host *Escherichia coli* is also predicted.

**Conclusions:**

The proposed model is an effective computational tool for predicting interactions between viruses and hosts effectively, and it has great potential for discovering novel hosts of viruses.

## Background

The two main components of human microbes are bacterial and viral communities, which play a vital role in human health and diseases. Bacterial communities have been proved to be associated with human diseases, including some skin conditions [1] and gastrointestinal diseases, such as inflammatory bowel disease [2], clostridium infection [3], and colorectal cancer [4]. Viral communities are also associated with diseases, such as periodontal disease [5] and antibiotic resistance [6]. Viruses are widespread in the environment and biological tissues, and they are the most abundant organisms on the planet [7]. Viruses cannot survive alone, they need to be parasitic in living cells to survive and produce offspring. Hosts infected by viruses include bacteria, archaea, eukaryotes, etc. Viruses produce DNA and proteins of offspring through hosts’ replication mechanism. In particular, prokaryotic viruses have a significant impact on human health and ecosystem dynamics. Describing interactions between viruses and hosts is important for understanding hosts’ effects on microbial communities.

The traditional approach for identifying viruses has been implemented by isolating from cultured host strains, because viruses are acquired from cultured host cells, we can directly know the host of the given virus. However, culturing a virus at a high enough concentration may be challenging in experiments because it may require appropriate growth conditions, such as temperature, growth media as well as robust growth of target host strains [8], which is usually difficult to achieve in experiments. The isolation method based on culturing bacteria is inefficient to identify viruses, and it only identifies relatively fewer viruses. Nowadays, discoveries of unknown viruses have been greatly accelerated by metagenomic shotgun sequencing, but unlike viral isolation, viral sequences assembled from metagenomics usually fails to directly obtain hosts infected by them. For example, crAssphage is a highly abundant human enterovirus, which may play an important role in the human intestinal tract, but the cultivation of crAssphage in the laboratory is still not achievable, so its hosts and biological function has not yet been identified [9]. As more and more metagenomic sequencing datasets are available, it is urgent to propose effective culture-free methods to identify new viruses and their hosts.

Recognizing hosts infected by viruses is important for understanding dynamics of viruses and their effects on microbial communities. Recently, some computational methods have been used to infer associations between viruses and hosts. Edwards [10] et al. introduced three types of virus-host association prediction methods, including sequence homology [6, 11, 12], abundance profile co-occurrence [13] and sequence composition [14–16]. As for virus-host association prediction methods based on sequence homology, homologies between new viruses and potential hosts are limited, because they depend on whether hosts of the query virus exist in the host genome database. Abundance profile method is based on co-variation, but significant co-variation does not necessarily represent real interaction. Because there is usually a time delay in dynamic interactions between viruses and hosts, many interactions depending on time-scale sampling may not be detected. Sequence composition is based on codon usage or short pairs of nucleotides (k-mers) shared by viruses and hosts to predict which hosts the virus infects. Ahlgren et al. proposed 11 measurements of oligonucleotide frequency (ONF) such as ${d}_2^{\ast }$ to calculate k-mers distances between viruses and hosts [17]. This method achieves good results in host prediction accuracy at the genus level, but less than 40% at the species level. In addition, previous human microbial community studies relied on independent bacterial and viral communities, i.e. they were divided into two separate network communities [2, 5], which could not capture complex dynamics of virus-host interactions.

In this paper, we propose a logistic matrix factorization algorithm based on integrating multi-information on the heterogeneous network to predict potential virus-host associations (ILMF-VH). The main differences from previous studies are that our proposed method combines information of three networks to form a virus-host heterogeneous network and applies similar network fusion (SNF) to integrate multiple host information for constructing the host-host similarity network. We used the benchmark data of viral and bacterial genomes in NCBI, and verified that ILMF-VH obtained best performance compared with recent five network-based methods under five-fold cross validation. Moreover, the host prediction accuracy is 63.66% which is 24.66 and 13.29% higher than two recently proposed virus-host association prediction methods respectively, and it is 0.49% higher than our previous approach [18]. In addition, the host of crAssphage inferred by our algorithm includes putative host *Bacteroides* obtained from previous studies [9, 19], and another potential host *Escherichia coli* is also suggested. Because previous studies have shown that *Escherichia coli* is associated with human intestinal diseases, such as diarrhea [20], our research indicates that crAssphage may be closely related to these diseases, and this proves that our approach is effective in predicting novel virus-host associations.

## Materials and methods

### Data sets

We used the data adopted by Ahlgren et al. which collected accession numbers and taxonomies of 1427 viruses and 31,986 hosts. For the initial analysis, we selected a subset including 352 viruses whose hosts were at strain level [17]. In addition, we downloaded the benchmark datasets provided by Edwards et al. including accession numbers and taxonomies of 820 viruses and 2699 hosts [21]. Based on accession numbers of viruses and hosts, we have written scripts to obtain their whole genome sequences from NCBI. In terms of each virus, their known virus-host associations are obtained through the ‘isolate host = ‘or ‘host = ‘fields in the viral annotation file. The genome of crAssphage in the human intestinal metagenomic is downloaded from NCBI and the accession number is JQ995537.1 [19].

### Methods

As for our model, the virus set and host set are represented by $V=\left\{{v}_1,{v}_2,\dots, {v}_{N_v}\right\}$ and $H=\left\{{h}_1,{h}_2,\dots, {h}_{N_h}\right\}$, where *N*_*v*_ and *N*_*h*_ represent the number of viruses and hosts, respectively. The associations between viruses and hosts are defined as an adjacency matrix $Y\in {R}^{N_v\times {N}_h}$, if a virus *v*_*i*_ is known to be associated with a host *h*_*j*_, then *y*_*ij*_ is set to 1; otherwise, *y*_*ij*_ is set to 0. In terms of elements in the adjacency matrix *Y*, the negative and positive interactions between viruses and hosts are represented by 0 and 1, respectively. In this work, firstly, we define a set of viruses which are positively related to hosts as ${V}^{+}=\left\{{v}_i|\sum \limits_{i=1}^{N_v}{y}_{ij}>0,\forall 1\le i\le {N}_v\right\}$, then a set of viruses which are negatively related to hosts is defined as *V*^−^ = *V*\*V*^+^. Next, a set of hosts which are positively related to viruses is defined as ${H}^{+}=\left\{{h}_j|\sum \limits_{j=1}^{N_h}{y}_{ij}>0,\forall 1\le j\le {N}_h\right\}$, and a set of hosts which are negatively related to viruses is defined as *H*^−^ = *H*\*H*^+^. Finally, similarities between viruses are calculated by oligonucleotide frequency (ONF) measures and expressed by ${S}^v\in {R}^{N_v\times {N}_v}$; similarities between hosts are calculated by integrating ONF measures and Gaussian interaction profile (GIP) kernel similarity based on SNF model, and expressed by ${S}^h\in {R}^{N_h\times {N}_h}$.

### Oligonucleotide frequency measures for viruses and hosts

Recently, dissimilarity measurements based on k-mer frequencies have been applied to infer relationships between genomic sequences [17]. Here, based on the hypothesis that similar viruses or hosts share similar k-mer patterns, we calculated k-mer similarities between viral genomic sequences to measure correlations between viruses. Similarly, k-mer similarities between hosts’ genomic sequences are calculated to measure correlations between hosts. According to previous research [17], ${d}_2^{\ast }$[22] has a good performance in calculating k-mer similarity and *k* is set to 6, so we calculate the distance between k-mer frequency vectors of each pair of viruses or hosts. Finally, the virus-virus similarity matrix *S*^*v*^ and the host-host similarity matrix *S*(*onf*)^*h*^ can be obtained.

### Gaussian interaction profile kernel similarity for hosts

Zou et al. [23] calculated the GIP kernel similarity between microbes based on the known disease-microbe association matrix and achieved good results. Apart from sequence similarities of hosts, based on the assumption that similar hosts exhibit similar patterns with viruses, we apply GIP kernel similarity to measure associations between hosts. There are two steps to calculate GIP kernel similarity. First, the interaction profile *IP*(*h*_*i*_) of host *h*_*i*_is the i-th column of the adjacency matrix *Y*, which is a binary relationship vector representing associations between a host *h*_*i*_and each virus. The GIP kernel similarity between host *h*_*i*_and *h*_*j*_ is calculated from their interaction profiles and defined as [24]:

1 $$ {S}^h\left({h}_i,{h}_j\right)=\exp \left(-{\gamma}_h{\left\Vert IP\left({h}_i\right)- IP\left({h}_j\right)\right\Vert}^2\right) $$

This is a kernel that represents the similarities between hosts. These kernels are called Gaussian kernels. The parameter *γ*_*h*_ is used to control the kernel bandwidth and defined as:

2 $$ {\gamma}_h={r}_h^{\prime }/\left(\ \frac{1}{N_h}{\sum}_{k=1}^{N_h}{\left\Vert IP\left({h}_k\right)\right\Vert}^2\ \right) $$

Here, *N*_*h*_ is the number of hosts. According to the previous study [25], we simply set ${r}_h^{\prime }$ to 1.

### Integrated similarity for hosts

The associations between hosts are measured by calculating ONF measures and GIP kernel similarity between hosts, respectively. Here, we introduce similar network fusion (SNF) [26] to integrate two host similarity networks. The SNF includes following three main steps. First, the edge weights of each host similarity network are represented by a *N*_*h*_ × *N*_*h*_ matrix *S*^*h*^, respectively. Then, as for each similarity network, a normalized weight matrix *p* can be obtained by the following formula [26]:

3 $$ {P}_{i,j}=\left\{\begin{array}{c}\frac{S\left(i,j\right)}{2{\sum}_{k\ne i}S\left(i,k\right)},j\ne i\\ {}\ \frac{1}{2},j=i\end{array}\right. $$

Here *S*(*i*, *j*) is the matrix element of *S*^*h*^. Then, *k* nearest neighbor (KNN) is used to measure the local relationship as follows:

4 $$ KNN\left(i,j\right)\left\{\begin{array}{c}\frac{S\left(i,j\right)}{\sum_{k\epsilon {N}_i}S\left(i,k\right)}, j\epsilon {N}_i\\ {}\ 0, otherwise\end{array}\right. $$

*N*_*i*_ represents the number of neighbors in the host. This method filters out low-similar edges.

Let *P*^(*v*)^ and *KNN*^(*v*)^ represent similar matrices of the above two hosts, respectively. The process of SNF is an iterative update of similarity matrices, which corresponds to each data type as follows [26]:

5 $$ {P}^{(v)}={KNN}^{(v)}\left(\frac{\sum_{k\ne v}{P}^{(k)}}{m-1}\right){\left({KNN}^{(v)}\right)}^T,v=1,2\dots, m $$

This step updates the matrix *P*^(*v*)^ when *m* parallel exchange diffusion processes are generated on *m* host networks. In this paper, we have two types of host similar matrices, so *m* is set to 2. The final similarity matrix that integrates all data types is defined as follows:

6 $$ P=\frac{1}{2}\left({P}^1+{P}^2\right) $$

### Construction of heterogeneous networks

The construction of heterogeneous networks is mainly divided into three steps. First, based on known virus-host associations, we can construct a virus-host relationship network, where nodes in the network include viruses and hosts and if a virus and a host are known to be related, their edge weights are set to 1, otherwise, they are set to 0. Then, we calculate similarities between viruses based on ONF measures to construct the virus network, and calculate similarities between hosts by integrating ONF measure and GIP kernel similarity based on SNF model to construct the host network. Finally, the virus network and the host network are connected through known virus-host associations to construct a heterogeneous network between viruses and hosts.

### Kernelized logistic matrix factorization

**Fig. 1:**
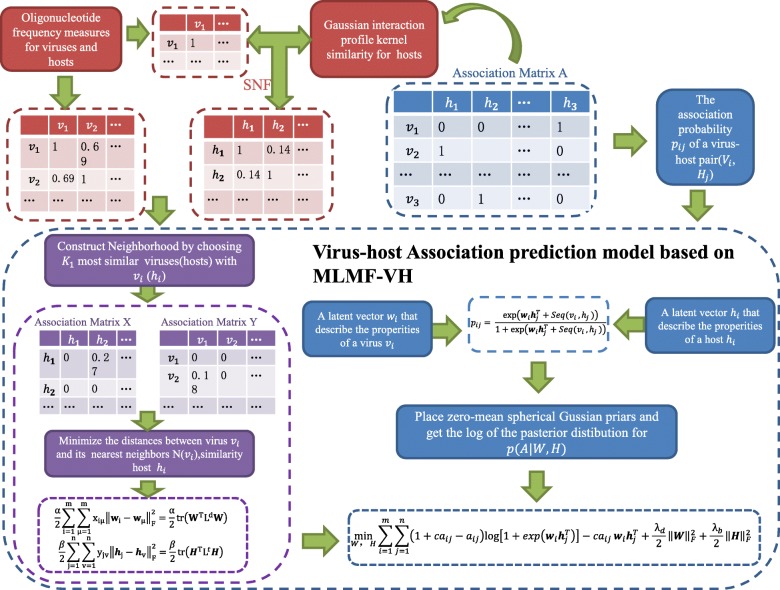
The flow chart of ILMF-VH model

We developed a kernelized logistic matrix factorization algorithm based on network similarity fusion for predicting virus-host associations, and the flowchart of ILMF-VH model is shown in the Fig. 1. First, the binary matrix *Y* is decomposed into $W\in {R}^{N_v\times k}$ and $\in {R}^{N_h\times k}$, so viruses and hosts are mapped to the shared potential low-dimensional space. *Seq*(*v*_*i*_, *h*_*j*_) represents the ONF similarity between each pair of virus and host, and we integrate this sequence similarity information into the associated probability *p*_*ij*_, which represents association probability of virus-host pair (*v*_*i*_, *h*_*j*_) and is defined as the logistic function:

7 $$ {p}_{ij}=\frac{\exp \left({\boldsymbol{w}}_i{\boldsymbol{h}}_j^T+ Seq\left({v}_i,{h}_j\right)\right)}{1+\exp \left({\boldsymbol{w}}_i{\boldsymbol{h}}_j^T+ Seq\left({v}_i,{h}_j\right)\right)} $$

It is hypothesized that known relationships between viruses and hosts provide useful information for virus-host association prediction. Current importance weighting methods have been proven to be effective for personalized recommendations and drug-target interaction predictions [27, 28]. We apply the weight constant c to control the level of importance between each known and unknown associations. According to previous studies, *c* is set to 5. The conditional probability of *Y* is defined as:

8 $$ p\left(Y|W,H\right)={\prod}_{i=1}^{N_v}{\prod}_{j=1}^{N_h}{p_{ij}}^{c{y}_{ij}}{\left(1-{p}_{ij}\right)}^{\left(1-{y}_{ij}\right)} $$

In this work, we also use the neighborhood regularization method to regularize the logistic matrix factorization algorithm [28]. The nearest neighbors of virus *v*_*i*_ and host *h*_*i*_ are defined as *N*(*v*_*i*_) ∈ *V*\*v*_*i*_ and *N*(*h*_*j*_) ∈ *H*\*h*_*i*_, *N*(*v*_*i*_) and *N*(*h*_*i*_) represent the *K*_1_ neighbors of the virus *v*_*i*_ or the host *h*_*i*_, respectively. *K*_1_ is set to 5 according to the experiment. The neighborhood information of viruses and hosts is represented by the adjacency matrices *A* and *B*, respectively. In terms of matrix *A*, if virus *v*_*m*_ ∈ *N*(*v*_*i*_),${a}_{im}={s}_{im}^v$, otherwise *a*_*im*_ = 0; in terms of matrix *B*, if host *h*_*n*_ ∈ *N*(*h*_*j*_),${b}_{jn}={s}_{jn}^h$, otherwise *b*_*jn*_ = 0 . The main purpose of virus-host association prediction is to minimize distances between *v*_*i*_/*h*_*i*_ and nearest neighbors *N*(*v*_*i*_)/*N*(*h*_*i*_). We should try to minimize the following objective formula:

9 $$ \frac{\alpha }{2}{\sum}_{i=1}^{N_v}{\sum}_{m=1}^{N_v}{a}_{im}{\left\Vert {w}_i-{w}_m\right\Vert}_F^2=\frac{\alpha }{2} tr\left({W}^T{L}^vW\right) $$

10 $$ \frac{\beta }{2}{\sum}_{i=1}^{N_h}{\sum}_{m=1}^{N_h}{b}_{jn}{\left\Vert {w}_j-{w}_n\right\Vert}_F^2=\frac{\beta }{2} tr\left({W}^T{L}^hW\right) $$

Where tr(∙) is the trace of the matrix, ${L}^v=\left({D}^v+\overset{\sim }{D^v}\right)-\left(A+{A}^T\right)$, and the diagonal element of *D*^*v*^ is ${D}_{ii}^v={\sum}_{m=1}^{N_v}{a}_{im}$; ${L}^h=\left({D}^h+\overset{\sim }{D^h}\right)-\left(B+{B}^T\right)$, the diagonal elements of *D*^*h*^ are ${D}_{jj}^h={\sum}_{n=1}^{N_h}{b}_{jn}$. Our goal is to find the minimum of the following objective functions:

11 $$ \underset{W,H}{\mathit{\min}}{\sum}_{i=1}^{N_v}{\sum}_{j=1}^{N_h}\left(1+{cy}_{ij}-{y}_{ij}\right)\mathit{\ln}\left[1+\mathit{\exp}\left({\boldsymbol{w}}_i{\boldsymbol{h}}_j^T\right)\right]-{cy}_{ij}{\boldsymbol{w}}_i{\boldsymbol{h}}_j^T+\frac{1}{2} tr\left[{\boldsymbol{W}}^T\left(\ {\lambda}_v\boldsymbol{I}+\alpha {\boldsymbol{L}}^v\right)\boldsymbol{W}\right]+\frac{1}{2} tr\left[{\boldsymbol{H}}^T\left(\ {\lambda}_h\boldsymbol{I}+\beta {\boldsymbol{L}}^h\right)\boldsymbol{H}\right] $$

Where ${\uplambda}_v=\frac{1}{\sigma_v^2},{\uplambda}_h=\frac{1}{\sigma_h^2}$, *σ*_*v*_ and *σ*_*h*_ are expressed as the variance of Gaussian distribution of viruses and hosts, respectively. ‖∙‖_F_ represents the Frobenius norm of the matrix, and *W* and *H* are randomly initialized using a Gaussian distribution with a mean of 0 and standard deviation of $\frac{1}{\sqrt{r}}$. We use the AdaGrad algorithm [29] to solve the optimization problem of Eq. (11).

When learning vectors *W* and *H*, vectors of the negative virus group or host group are learned only based on negative associations in the training process. However, some unknown virus-host associations may exist potential correlations. Based on previous studies, we replaced the vector of a negative virus/host with a linear combination of its neighbors in the positive set [28]. Here, we build *K*_2_ nearest neighbor sets for each virus and host separately and *K*_2_ is set to 5, according to the experimental study. We use *N*^+^(*v*_*i*_)/*N*^+^(*h*_*j*_) to express *K*_2_ nearest neighbors of *v*^*i*^ ∈ *V*^−^/*h*_*j*_ ∈ *H*^−^ in *V*^+^/*H*^+^ . Therefore, ***w***_*i*_ and ***h***_***j***_ in Eq. (7) are corrected to:

12 $$ {\overset{\sim }{\boldsymbol{w}}}_i=\left\{\begin{array}{c}{\boldsymbol{w}}_i\  if\ {v}_i\in {V}^{+}\\ {}\ \\ {}\ \frac{1}{\sum_{\mu \in {N}^{+}\left({v}_i\right)}{s}_{im}^v}{\sum}_{m\in {N}^{+}\left({v}_i\right)}{s}_{im}^v{\boldsymbol{w}}_m\  if\ {v}_i\in {V}^{-}\ \end{array}\right. $$

13 $$ {\overset{\sim }{\boldsymbol{h}}}_j=\left\{\begin{array}{c}{\boldsymbol{h}}_j\  if\ {h}_j\in {H}^{+}\\ {}\ \\ {}\ \frac{1}{\sum_{v\in {N}^{+}\left({h}_j\right)}{s}_{jn}^h}{\sum}_{n\in {N}^{+}\left({h}_j\right)}{s}_{jn}^h{\boldsymbol{h}}_n\  if\ {h}_j\in {H}^{-}\ \end{array}\right. $$

### Evaluation metrics

Based on the heterogeneous network constructed by the above method, we compare the AUC [30] and AUPR [31] of ILMF-VH and recent five network-based algorithms by five times five-fold cross-validation to evaluate their performances. Then, based on previous studies [10, 17], we evaluated our virus-host association prediction methods by host prediction accuracy on a benchmark dataset including 820 viruses genomes. The host prediction accuracy refers to the percentage of the virus which is predicted to have the same host taxonomy level as known hosts of the query virus.

## Results and discussion

### Performance evaluation of different based-network methods

In order to assess the performance of our model, we trained datasets including 352 viruses and 71 hosts to obtain model parameters and tested our model on benchmark datasets including 820 viruses and 2699 hosts. In addition, we compare ILMF-VH model with five recently proposed network-based methods (LMFH-VH [18], NetLapRLS [29], KBMF2K [32], BLM -NII [33], CMF [34]) through five-fold cross-validation in the dataset containing 352 viruses. In each round of five-fold cross-validation, one-fifth of the virus-host associations are set to test data, and corresponding elements in the adjacency matrix *Y* are set to 0, the other four subsets are used as training data. It should be noted that in each round of five-fold cross-validation experiment, when virus-host relationships are set to 0, the *Y* matrix has been changed, so each time we need to recalculate GIP kernel similarities between hosts, and then kernel similarities can be fused with ONF similarities of hosts by applying SNF model to obtain updated host-host similarities. In addition, according to previous studies [28, 32–35], the range of parameter settings for each method is shown in the Table 1. Here, we use a random search strategy [36] for each model to select optimal parameters.

**Table 1:** Experimental setup for the five network-based modeling process

Method	Parameter	Range of Parameter
ILMF/LMFH-VH	*α*	{2^−5^, 2^−4^…, 2^2^}
	*β*	{2^−5^, 2^−4^…, 2^0^}
	*λ*_*v*_, *λ*_*h*_	{2^−5^, 2^−4^…, 2^1^}
	*γ*	{2^−3^, 2^−2^…, 2^0^}
	*k*	{20,40,60…100}
KBMF	r	{50,100}
NetLapRLS	gamma_d	{10^−3^, 10,^−2^…, 2^0^}
	beta_d, beta_t	{2^−3^, 2,^−2^…, 2^0^}
BLM-NII	combination weight α	{0,0.1,0.2, …, 0.9,1}
CMF	*k*	{50,100}
	regularization coefficient lambda_l	{2^−2^, …2^1^}
	lambda_d, lambda_t	{2^−3^, 2^−2^, …2^5^}

Table 2 shows the AUC values and AUPR values obtained by six methods in the data sets including 352 viruses. The results showed that ILMF-VH achieved the best performance and AUC value and AUPR value are 0.9202 and 0.6243, respectively. This result demonstrates the effectiveness of our model in virus-host association prediction.

**Table 2:** The AUC and AUPR obtained by ILMF-VH and other five network-based methods

AUC						
	ILMF-VH	LMFH-VH	KBMF	BLM-NII	NetLapRLS	CMF
352 virus	**0.9202**	0.8568	0.7934	0.8201	0.6711	0.8286
AUPR						
	ILMF-VH	LMFH-VH	KBMF	BLM-NII	NetLapRLS	CMF
352 virus	**0.6243**	0.5560	0.3408	0.10054	0.2749	0.3100

### Sensitivity analysis of parameter values

As seen in Additional file 1: Figure S1-Figure S4, these figures show AUPR values obtained by ILMF-VH model corresponding to different parameter settings. We also tested effects of different *K* value (the number of neighbors of KNN) of SNF model on AUPR values (Additional file 1: Figure S5). So, we mainly analyze five parameters of ILMF-VH and the number of neighbors *K* of SNF model.

More specificity, we analyse the change trend of AUPR values with different factorization factor *k* used for matrix factorization. As shown in Additional file 1: Figure S1, the optimal value of *k* is 100 and average AUPR value of ILMF-VH is 0.6305 under five-fold cross validation. In addition, we also study impacts of regularization parameters *α* and *β* used for neighborhood smoothing in the prediction procedure. Additional file 1: Figure S2 shows the change trend of AUPR values under different *α* and *β*. The optimal values ​​of *α* and *β* are 0.0625 and 0.25, respectively. When *α* > 0.0625 and *β* > 0.25, corresponding AUPR values begin to decrease. These results emphasize that neighbor regularization has a certain impact on the virus-host prediction model. Moreover, we also analyse effects of *λ* on the prediction procedure. Here, $\lambda ={\lambda}_v=\frac{1}{\sigma_v^2}={\lambda}_h=\frac{1}{\sigma_h^2}$, *σ*_*v*_ and *σ*_*h*_ represent the variance of Gaussian distribution of viruses and hosts, respectively. As shown in Additional file 1: Figure S3, the AUPR value becomes larger gradually with the increase of *λ*, and when *λ* equals 2, AUPR reaches optimal value. Additional file 1: Figure S4 shows the variation trend of AUPR when learning rate parameters *γ* is set to different values. When *γ* equals 0.25, AUPR takes the optimal value; when γ increases, the AUPR value begins to decrease, so *γ* is set to 0.25. Furthermore, we also analyzed influences of different neighbor parameter *K* of SNF model on AUPR values. As shown in Additional file 1: Figure S5, the AUPR value reaches the optimal value when *K* is set to 5; when *K* increases again, the AUPR value begins to decrease, so the optimal value of *K* is 5.

### Comparison of ILMF-VH and previous virus-host prediction studies

**Fig. 2:**
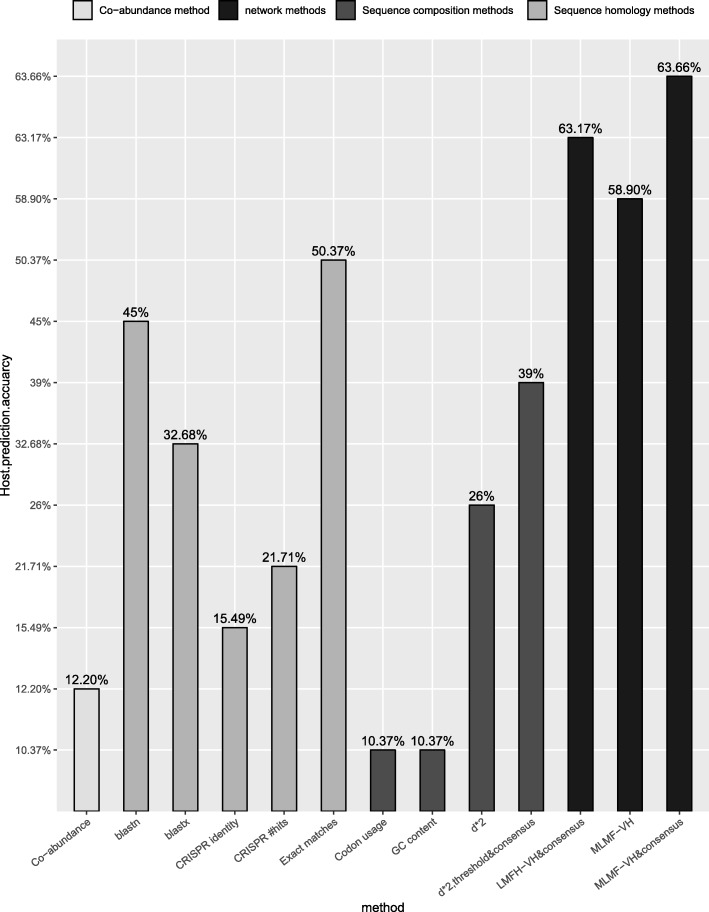
The host prediction accuracy of four types of methods for benchmark datasets including 820 viruses and 2699 hosts

In this work, we apply the ILMF-VH method to the benchmark dataset including 820 viruses and 2699 complete bacterial genomes. First, we calculate scores between each virus and candidate hosts. The higher the predicted score, the more likely the virus is infected by the host. Here, the highest ranked host is identified as the predicted result of the given virus, and if the predicted host is the same as known host of the given virus at the species level, the predicted host is considered as a correct one. Figure 2 shows the host prediction accuracy of four types of methods include abundance profile co-occurrence, sequence homology, sequence composition, and network-based. The result shown that ILMF-VH achieved the highest host prediction accuracy (58.90%) compared with other three types of methods.

In order to further improve the host prediction accuracy, we apply a consensus method [17] to our method. We believe that the most frequent host species in the top *n* predicted hosts of a virus can be classified as the host taxon of the given virus. The prediction accuracy is highest at *n* = 5, therefore, we selected the most frequent classification among the top 5 hosts as the host taxon of the query virus. As shown in Fig. 2, when a consensus strategy is applied to our model, the host prediction accuracy can be increased to 63.66%, which is 24.66%, 13.29 and 0.49% higher than three proposed virus-host prediction methods [10, 17, 18], respectively.

As for the general situation, when a new virus lacks host information, we can use the ILMF-VH method to predict its potential hosts. First, we constructed a virus-host network based on known virus-host associations; then GIP kernel similarities between hosts based on known virus-host associations can be calculated, and these GIP kernel similarities and ONF similarities of hosts are integrated through SNF model, so the host similarity network can be constructed. At the same time, we can calculate ONF similarities of whole genome sequences between the new virus and other viruses in the virus-host association network, so the virus similarity network can be established; finally, the ILMF-VH method is executed on the virus-host heterogeneous network, thereby the predicted scores between the new virus and all candidate hosts can be obtained.

### Case study

In this work, we evaluated the performance of ILMF-VH model through case studies. In a recent study, Dutil et al. used viral metagenomic sequencing data in human fecal samples to find [19] a highly abundant phage crAssphage and utilized co-occurrence profile of crAssphage and 404 potential human intestinal bacterial hosts from 151 human gut genomes in the human microbiome program to predict hosts of crAssphage, their results indicates that the host of crAssphage belongs to *Bacteroidetes*. At the same time, Ahlgren et al. [17] predicted potential hosts of crAssphage based on sequence similarities between crAssphage and candidates hosts; WANG et al. [9] used the Markov random field integration network to predict potential hosts of crAssphage. They all suggested that bacteria belonging to *Bacteroidetes* are the host of crAssphage. According to previous study [19], crAssphage is a virus that is widely found in the human gut genome, but we know very little about its biological significance and hosts of crAssphage, due to the difficulty of culturing crAssphage. Different methods have been proposed to predict hosts of the given virus, our information integration algorithm validates the host of crAssphage which was found in previous studies and also predicts another potential host *Escherichia coli*.

As for each virus, the candidate host is ordered according to predicted association scores obtained by ILMF-VH algorithm. In this paper, we assume that if the known candidate host of a virus *v*_*j*_ is *h*_*i*_, another new host *h*_*k*_ at the same taxon level as the host *h*_*i*_ may be a potential host of the virus *v*_*j*_. At the same time, the higher the predicted score of the candidate host *h*_*k*_, the more likely it is to have a potential correlation with the query virus. In the case study, we added the whole genome sequence of crAssphage to the similarity network containing 820 viruses, that is, similarities between the crAssphage and 820 virus sequences can be calculated based on ONF measurement, thus a new virus-virus similarity network can be constructed. Apart from that, we also add links between crAssphage and 2699 hosts to the virus-host network to build a new virus-host association network. Based on ONF measurement and known associations between viruses and hosts, we used our algorithm to obtain predicted scores between crAssphage and 2699 candidate hosts.

Our approach supports the previous conclusion that candidate hosts belonging to *Bacteroides* are potential hosts of crAssphage. As for the top 50 predicted hosts of crAssphage, there were three hosts belonging to phylum *Bacteroidetes* and were ranked 4th, 44th and 50th: *Cardinium endosymbiont of Encarsia pergandiella*, *Weeksella virosa*, and *Tannerella forsythia*. Our prediction model also inferred that *Escherichia coli* belonging to phylum *Proteobacteria* is the potential host of crAssphage, and *Escherichia coli* ranks highest among 2699 hosts. A possible explanation for its highest predicted score is that the alignment-free similarity score between crAssphage and *Escherichia coli* is 0.6568, which is higher than the average score (0.6096) between the virus and all candidate hosts. Therefore, sequence alignment is an important part of extracting virus-host association signal, and it provides an efficient contribution indicator for this prediction result.

Our algorithm predicted host of crAssphage based on the metagenomics sequencing data, which is identical to the putative host at phylum level in previous studies. In addition, another potential host *Escherichia coli* is also inferred. Recent studies have shown that [20] most *Escherichia coli* strains grow harmlessly in the gut and rarely cause diseases in healthy individuals. However, many pathogenic strains can cause diarrhea or extraintestinal disease in both healthy and immunocompromised individuals. Our experimental results suggest that crAssphage may play an important role in these diseases. In general, our algorithmic model is effective in predicting potential hosts of new viruses.

## Conclusion and outlook

Viral infection usually results in changes in the ecosystem function of host cells. Virus-host association studies can reveal complex virus-host network interactions and are important for understanding of microorganism diversity. Despite this, although some methods for virus-host association prediction have been proposed, the host prediction accuracy at the species level cannot be achieved very well and these methods need to be improved.

We present an effective method ILMF-VH for predicting virus-host associations. We performed the best performance compared to recent five network-based methods by five-fold cross-validation. Secondly, we compared the host prediction accuracy with several recently proposed virus-host association prediction methods [10, 17]. Our method obtained the highest host prediction accuracy (63.66%). Finally, we analyzed our method’s abilities to predict potential hosts for the given virus. As for the crAssphage, our predicted hosts are corresponding to previous studies, and predicted another host *Escherichia coli* is associated with intestinal diseases. In general, it is important to study virus-host associations. Our research not only has potential to predict hosts of viruses, but also can be applied to predict virus-host associations.

Although some results have been achieved so far, there are still some problems that can be further studied in the future. First, the biology characteristics of viruses and hosts are abundant and varied. Apart from whole genome sequences, protein, amino acid, abundance profile and other related information might also have contribution to the prediction model. It needs further research to study what information provides a reliable basis for virus-host association prediction, and extracting appropriate characteristics of viruses and hosts are important for predicting results. Here, we integrate genome sequence information and known virus-host associations. In the future researches, we will consider adding different information sources of viruses and hosts to analyze impacts of different characteristics on prediction results.

## Supplementary information

**Additional file 1: Figure S1.** The trend chart of AUPR values vary with the factorization factor *k*. **Figure S2.** The trend chart of AUPR values vary with regularization parameters *α* and *β*. **Figure S3.** The trend chart of AUPR values vary with the inverse of the variance *λ*. **Figure S4.** The trend chart of AUPR values vary with the learning rate parameter *γ*. **Figure S5.** The trend chart of AUPR values vary with the neighbor number parameter *K*.

## Data Availability

Our dataset is primarily derived from virus-host metagenomic sequencing data and known virus-host associations in NCBI. There is also the sequence of crAssphage in the human gut metagenomic. The data and code for this article is available at https://github.com/liudan111/ILMF-VH.git.

## References

[1] Hannigan GD, Grice EA (2013). Microbial ecology of the skin in the era of metagenomics and molecular microbiology. Cold Spring Harb Perspect Med.

[2] Norman JM, Handley SA, Baldridge MT, Droit L, Liu CY, Keller BC, Kambal A, Monaco CL, Zhao G, Fleshner P (2015). Disease-specific alterations in the enteric virome in inflammatory bowel disease. Cell.

[3] Seekatz AM, Rao K, Santhosh K, Young VB (2016). Dynamics of the fecal microbiome in patients with recurrent and nonrecurrent Clostridium difficile infection. Genome Med.

[4] Zackular JP, Rogers MA, MTt R, Schloss PD (2014). The human gut microbiome as a screening tool for colorectal cancer. Cancer Prev Res (Phila).

[5] Ly M, Abeles SR, Boehm TK, Robles-Sikisaka R, Naidu M, Santiago-Rodriguez T, Pride DT (2014). Altered oral viral ecology in association with periodontal disease. MBio.

[6] Modi SR, Lee HH, Spina CS, Collins JJ (2013). Antibiotic treatment expands the resistance reservoir and ecological network of the phage metagenome. Nature.

[7] Delwart EL (2007). Viral metagenomics. Rev Med Virol.

[8] Akhter S, Aziz RK, Edwards RA (2012). PhiSpy: a novel algorithm for finding prophages in bacterial genomes that combines similarity- and composition-based strategies. Nucleic Acids Res.

[9] Wang W, Ren J, Ahlgren NA, et al. A network-based integrated framework for predicting virus-host interactions with applications[J]. bioRxiv. 2018. 10.1101/505768.

[10] Edwards RA, McNair K, Faust K, Raes J, Dutilh BE, Smith M (2016). Computational approaches to predict bacteriophage–host relationships. FEMS Microbiol Rev.

[11] Horvath P, Barrangou R (2010). CRISPR/Cas, the immune system of bacteria and archaea. Science.

[12] Roux S, Enault F, Hurwitz BL, Sullivan MB (2015). VirSorter: mining viral signal from microbial genomic data. PeerJ.

[13] Nielsen HB, Almeida M, Juncker AS, Rasmussen S, Li J, Sunagawa S, Plichta DR, Gautier L, Pedersen AG, Le Chatelier E (2014). Identification and assembly of genomes and genetic elements in complex metagenomic samples without using reference genomes. Nat Biotechnol.

[14] Roux S, Hallam S J, Woyke T, et al. Viral dark matter and virus–host interactions resolved from publicly available microbial genomes. Elife. 2015;4:e08490. 10.7554/eLife.08490 PMC4533152 26200428

[15] Marcais G, Kingsford C (2011). A fast, lock-free approach for efficient parallel counting of occurrences of k-mers. Bioinformatics.

[16] Pride DT, Wassenaar TM, Ghose C, Blaser MJ (2006). Evidence of host-virus co-evolution in tetranucleotide usage patterns of bacteriophages and eukaryotic viruses. BMC Genomics.

[17] Ahlgren NA, Ren J, Lu YY, Fuhrman JA, Sun F (2017). Alignment-free $d_2^*$ oligonucleotide frequency dissimilarity measure improves prediction of hosts from metagenomically-derived viral sequences. Nucleic Acids Res.

[18] Liu D, Hu X, Jiang X (2018). Virus-host association prediction by using Kernelized logistic matrix factorization on heterogeneous networks. 2018 IEEE International Conference on Bioinformatics and Biomedicine (BIBM).

[19] Dutilh BE, Cassman N, McNair K, Sanchez SE, Silva GG, Boling L, Barr JJ, Speth DR, Seguritan V, Aziz RK (2014). A highly abundant bacteriophage discovered in the unknown sequences of human faecal metagenomes. Nat Commun.

[20] Gomes TA, Elias WP, Scaletsky IC, Guth BE, Rodrigues JF, Piazza RM, Ferreira LC, Martinez MB (2016). Diarrheagenic Escherichia coli. Braz J Microbiol.

[21] Edwards RA, McNair K, Faust K, Raes J, Dutilh BE (2016). Computational approaches to predict bacteriophage-host relationships. FEMS Microbiol Rev.

[22] Reinert G (2009). Alignment-free sequence comparison (I): statistics and power. J Comput Biol.

[23] Zou S, Zhang J, Zhang Z (2017). A novel approach for predicting microbe-disease associations by bi-random walk on the heterogeneous network. PLoS One.

[24] van Laarhoven T, Nabuurs SB, Marchiori E (2011). Gaussian interaction profile kernels for predicting drug-target interaction. Bioinformatics.

[25] Huang ZA, Chen X, Zhu Z, et al. PBHMDA: path-based human microbe-disease association prediction. Front Microbiol. 2017;8:233. 10.3389/fmicb.2017.00233 PMC5319991 28275370

[26] Wang B, Mezlini AM, Demir F, Fiume M, Tu Z, Brudno M, Haibe-Kains B, Goldenberg A (2014). Similarity network fusion for aggregating data types on a genomic scale. Nat Methods.

[27] Hu Y, Koren Y, Volinsky C. Collaborative filtering for implicit feedback datasets. Eighth IEEE International Conference on Data Mining. Pisa: IEEE; 2008:263–272.

[28] Liu Y, Wu M, Miao C, Zhao P, Li XL (2016). Neighborhood regularized logistic matrix factorization for drug-target interaction prediction. PLoS Comput Biol.

[29] Duchi J, Hazan E, Singer Y. Adaptive subgradient methods for online learning and stochastic optimization. J Mach Learn Res. 2011;12(Jul):2121–59.

[30] Hanley JA, McNeil BJ (1982). The meaning and use of the area under a receiver operating characteristic (ROC) curve. Radiology.

[31] Davis J, Goadrich M. The relationship between precision-recall and ROC curves: Proceedings of the 23rd international conference on Machine learning. New York: ACM; 2006. p. 233–240.

[32] Gonen M (2012). Predicting drug-target interactions from chemical and genomic kernels using Bayesian matrix factorization. Bioinformatics.

[33] Mei JP, Kwoh CK, Yang P, Li XL, Zheng J (2013). Drug-target interaction prediction by learning from local information and neighbors. Bioinformatics.

[34] Zheng X, Ding H, Mamitsuka H, et al. Collaborative matrix factorization with multiple similarities for predicting drug-target interactions: Proceedings of the 19th ACM SIGKDD international conference on Knowledge discovery and data mining. Chicago:ACM; 2013. p. 1025–1033.

[35] Xia Zheng, Wu Ling-Yun, Zhou Xiaobo, Wong Stephen TC (2010). Semi-supervised drug-protein interaction prediction from heterogeneous biological spaces. BMC Systems Biology.

[36] Bergstra JBY. Random search for hyper-parameter optimization. J Mach Learn Res. 2012;13(Feb).

