# Supplementary material for: Predicting virus-host association by Kernelized logistic matrix factorization and similarity network fusion
Source: BMC Bioinformatics. 2019 Dec 2;20(Suppl 16):594. doi: 10.1186/s12859-019-3082-0 (PMC6886165; doi:10.1186/s12859-019-3082-0)
Supplement: Supplementary file 1 — Additional file 1: Figure S1. The trend chart of AUPR values vary with the factorization factor k. Figure S2. The trend chart of AUPR values vary with regularization parameters α and β. Figure S3. The trend chart of AUPR values vary with the inverse of the variance λ. Figure S4. The trend chart of AUPR values vary with the learning rate parameter γ. Figure S5. The trend chart of AUPR values vary with the neighbor number parameter K. [file 12859_2019_3082_MOESM1_ESM.docx]

Supporting material for: Kernelized Logistic Matrix Factorization based on Similarity Network Fusion for Predicting Virus-host Association

Dan Liu^1, 2^, Yingjun Ma^1,2^, Xingpeng Jiang^1,2*^ and Tingting He^1,2*^

^1^School of Computer, Central China Normal University, Wuhan, Hubei, China

^2^Hubei Provincial Key Laboratory of Artificial Intelligence and Smart Learning，Central China Normal University, Wuhan, Hubei, China

^*^Corresponding Author: Xingpeng Jiang, xpjiang@mail.ccnu.edu.cn

Tingting He, tthe@mail.ccnu.edu.cn

Email addresses:

DL: [liudan@mails.ccnu.edu.cn](mailto:liudan@mails.ccnu.edu.cn)

YM: yingjunma@mails.ccnu.edu.cn

XH: [huxiaohua@mail.ccnu.edu.cn](mailto:huxiaohua@mail.ccnu.edu.cn)

TH: tthe@mail.ccnu.edu.cn

XJ: xpjiang@mail.ccnu.edu.cn

**Figure S1. The trend chart of AUPR values vary with the factorization factor $\boldsymbol{k}$.**


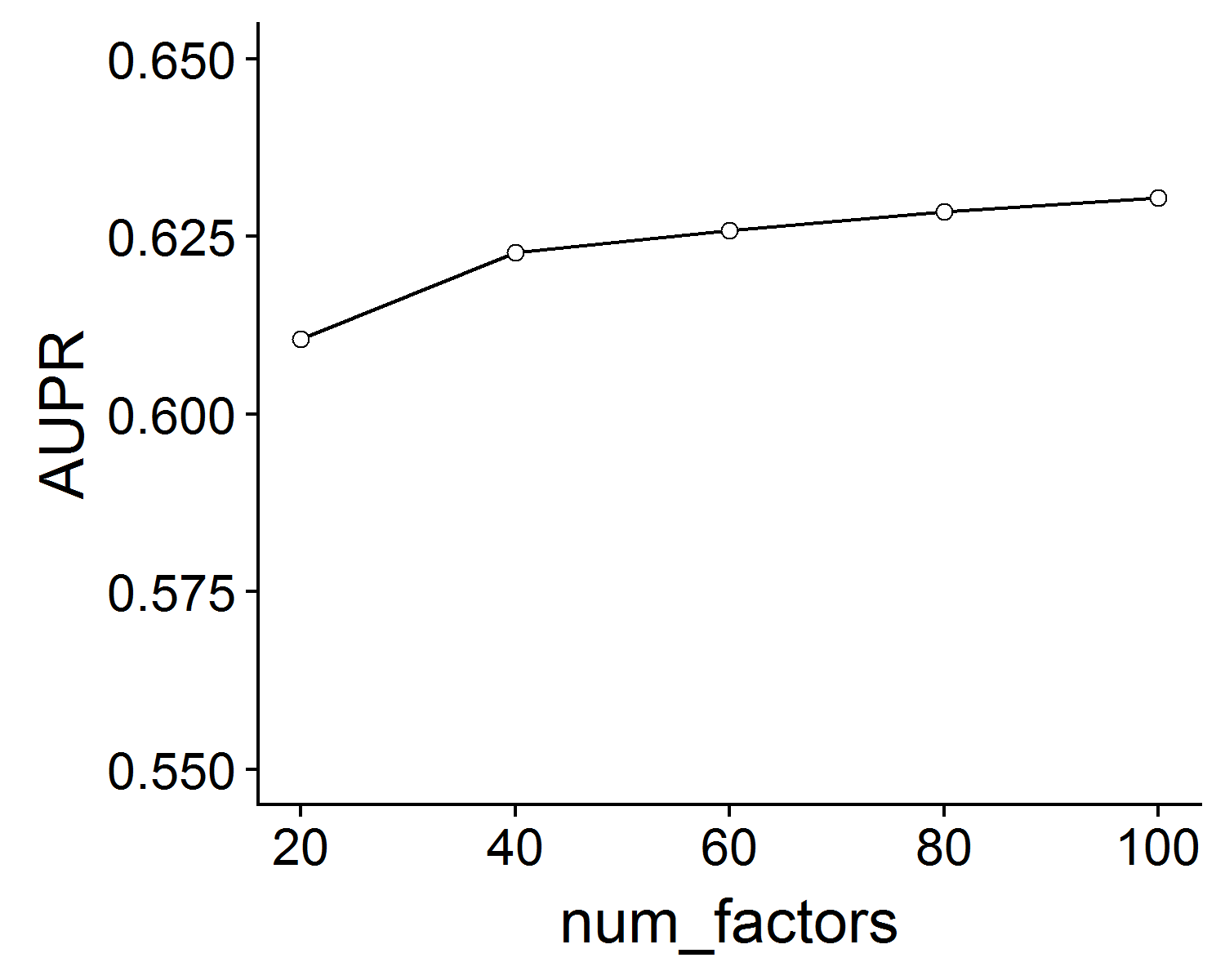


**Figure S2. The trend chart of AUPR values vary with regularization parameters $\boldsymbol{\alpha}$ and $\boldsymbol{\beta}$.**

**
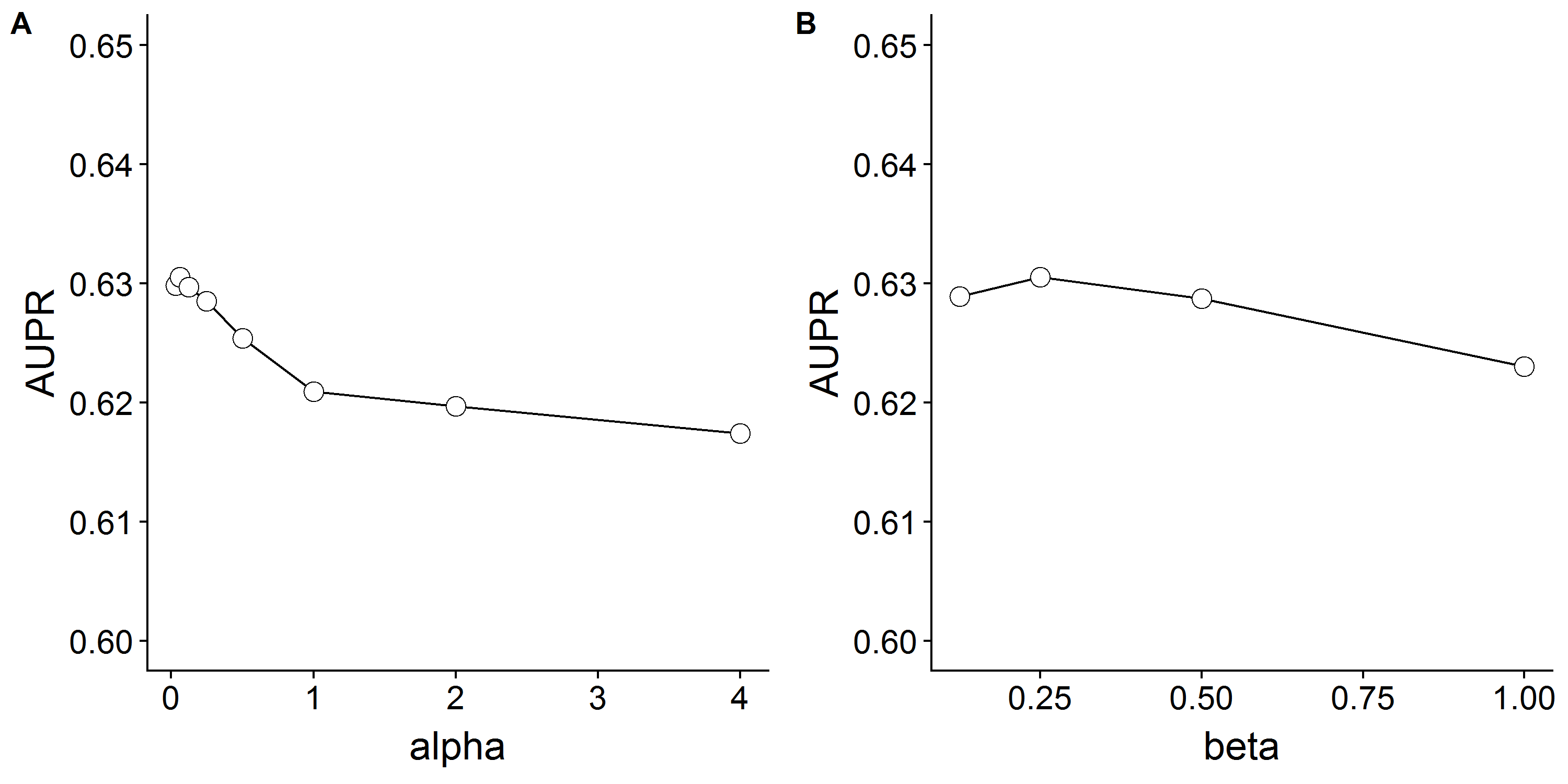
**

**Figure S3. The trend chart of AUPR values vary with the inverse of the variance $\boldsymbol{\lambda}$.**

**
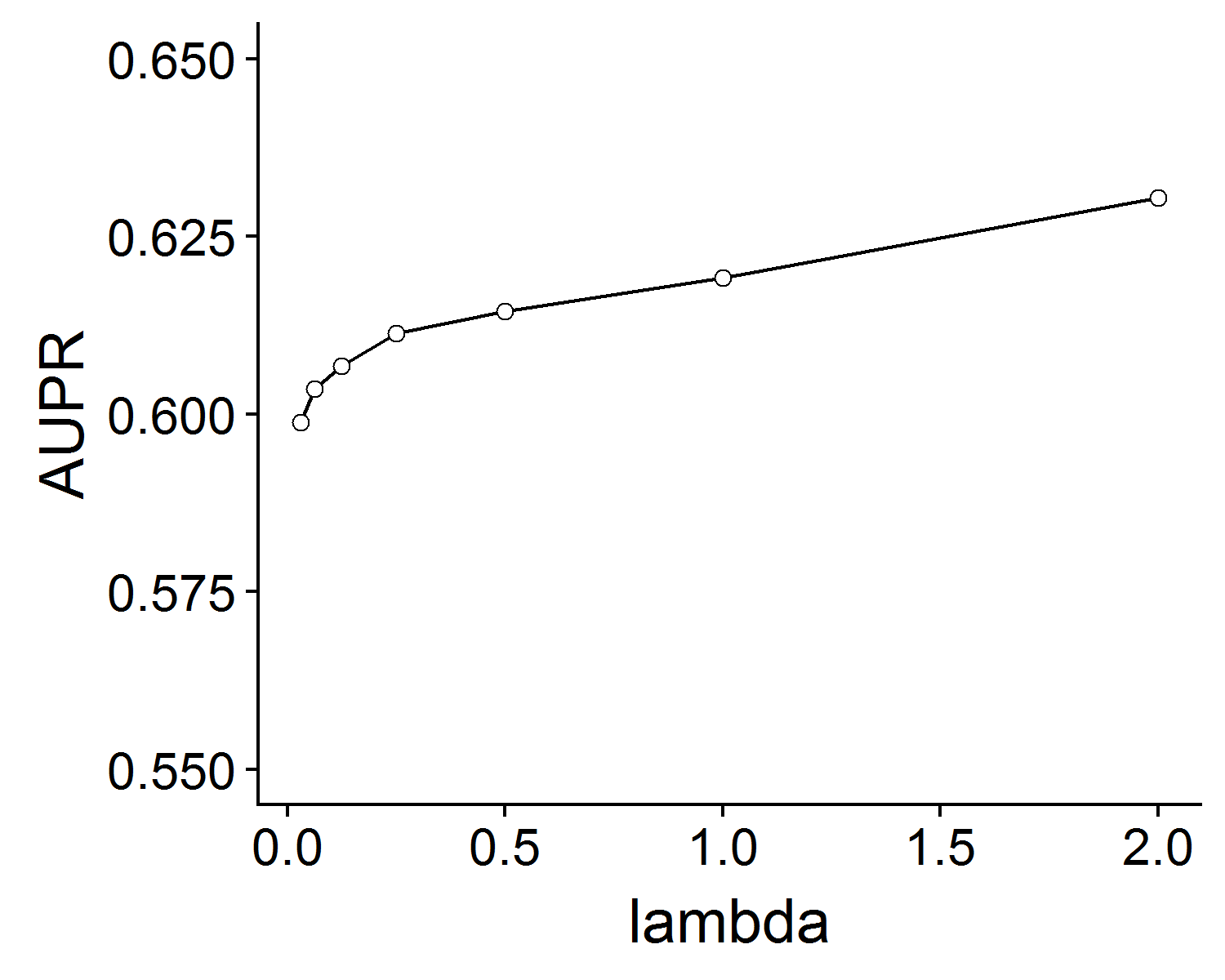
**

**Figure S4. The trend chart of AUPR values vary with the learning rate parameter $\boldsymbol{\gamma}$.**

**
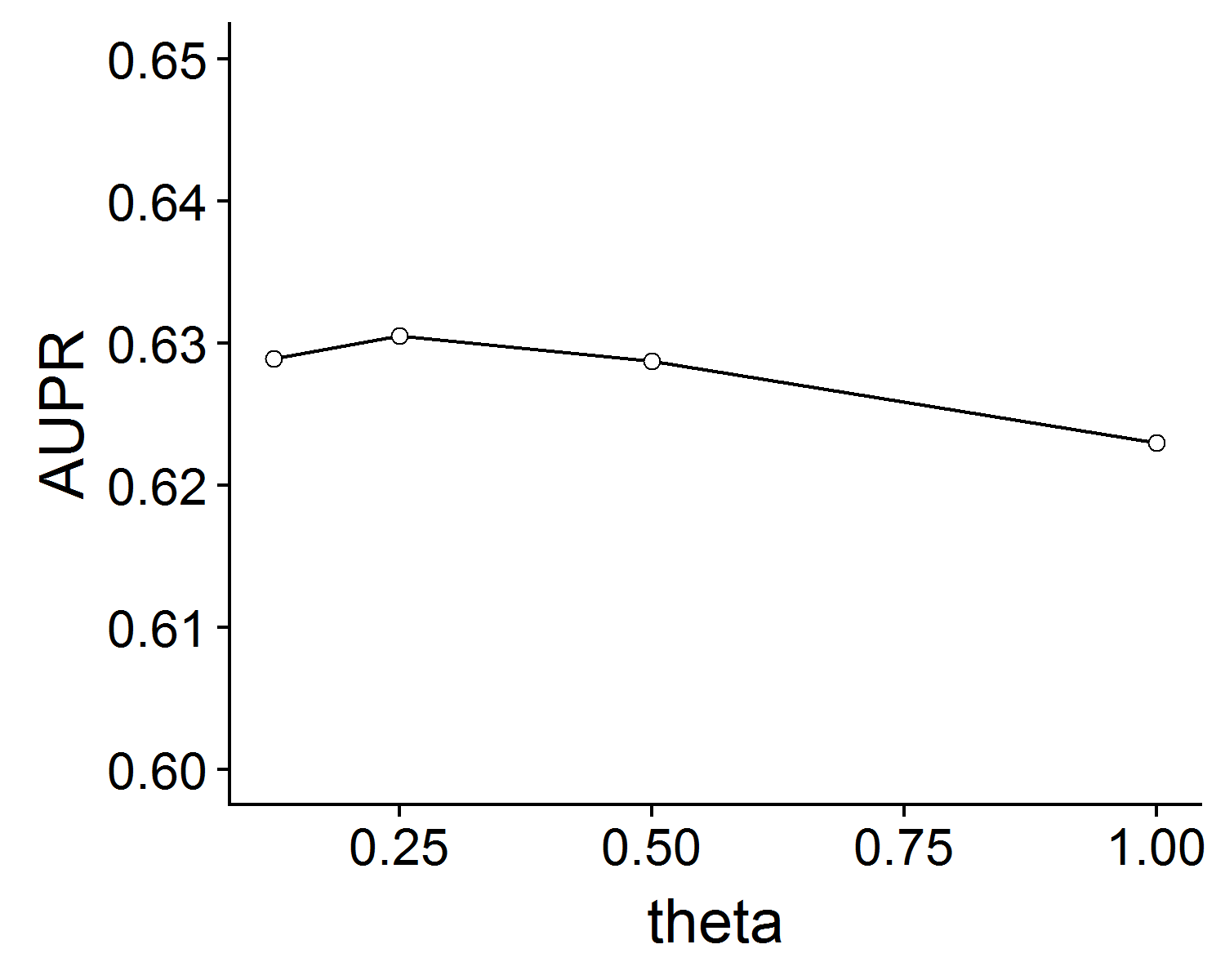
**

**Figure S5. The trend chart of AUPR values vary with the neighbor number parameter $\boldsymbol{K}$.**

**
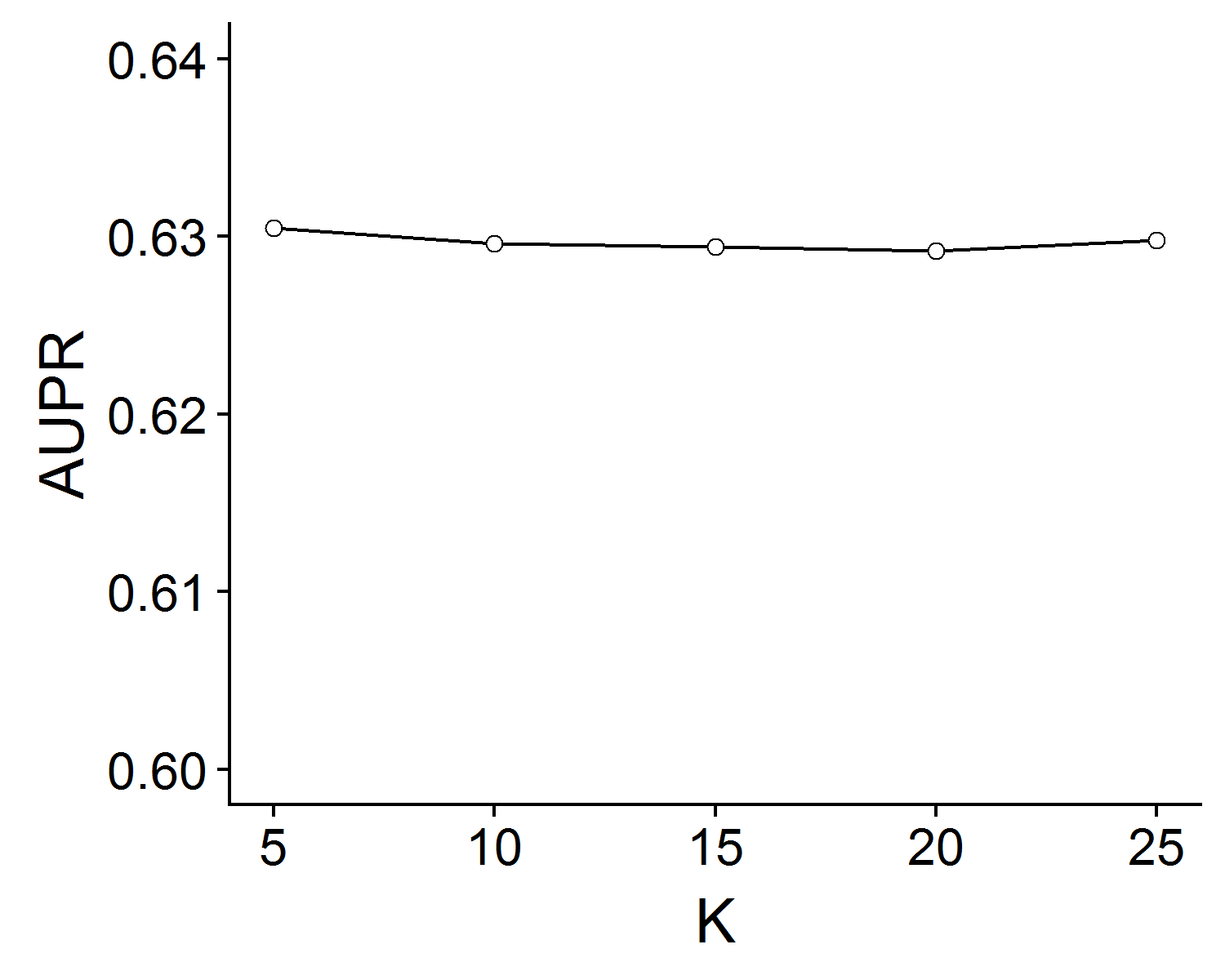
**
